# New approach for fish breeding by chemical mutagenesis: establishment of TILLING method in fugu (*Takifugu rubripes*) with ENU mutagenesis

**DOI:** 10.1186/1471-2164-14-786

**Published:** 2013-11-13

**Authors:** Miwa Kuroyanagi, Takashi Katayama, Tadashi Imai, Yoshihisa Yamamoto, Shin-ichi Chisada, Yasutoshi Yoshiura, Tomokazu Ushijima, Tomonao Matsushita, Masashi Fujita, Aoi Nozawa, Yuzuru Suzuki, Kiyoshi Kikuchi, Hiroyuki Okamoto

**Affiliations:** 1 Graduate School of Agricultural and Life Sciences, The University of Tokyo, 2971-4 Bentenjima, Maisaka, Hamamatsu, Shizuoka 431-0214, Japan; 2 Yashima station, Stock Enhancement and Management Division, National Research Institute of Fisheries and Enhancement of Inland Sea, Fisheries Research Agency, 243 Yashima-higashi, Takamatsu, Kagawa 761-0111, Japan; 3 Aquatic Animal Health Division, National Research Institute of Aquaculture, Fisheries Research Agency, 224-1 Hiruta, Tamaki, Mie 519-0423, Japan; 4 Faculty of Agriculture, Kyusyu University, 6-10-1 Hakozaki, Higashi-ku, Fukuoka 812-8581, Japan; 5 Aquaculture Technology Division, National Research Institute of Aquaculture, Fisheries Research Agency, 422-1 Nakatsuhamaura, Minami-ise, Mie 516-0193, Japan

**Keywords:** TILLING, Fugu, ENU, HRM, Myostatin, Mutagenesis, Fish breeding

## Abstract

**Background:**

In fish breeding, it is essential to discover and generate fish exhibiting an effective phenotype for the aquaculture industry, but screening for natural mutants by only depending on natural spontaneous mutations is limited. Presently, reverse genetics has become an important tool to generate mutants, which exhibit the phenotype caused by inactivation of a gene. TILLING (Targeting Induced Local Lesions INGenomes) is a reverse genetics strategy that combines random chemical mutagenesis with high-throughput discovery technologies for screening the induced mutations in target genes. Although the chemical mutagenesis has been used widely in a variety of model species and also genetic breeding of microorganisms and crops, the application of the mutagenesis in fish breeding has been only rarely reported.

**Results:**

In this study, we developed the TILLING method in fugu with ENU mutagenesis and high-resolution melting (HRM) analysis to detect base pair changes in target sequences. Fugu males were treated 3 times at weekly intervals with various ENU concentrations, and then the collected sperm after the treatment was used to fertilize normal female for generating the mutagenized population (F_1_). The fertilization and the hatching ratios were similar to those of the control and did not reveal a dose dependency of ENU. Genomic DNA from the harvested F_1_ offspring was used for the HRM analysis. To obtain a fish exhibiting a useful phenotype (e.g. high meat production and rapid growth), fugu myostatin (*Mstn*) gene was examined as a target gene, because it has been clarified that the *mstn* deficient medaka exhibited double-muscle phenotype in common with *MSTN* knockout mice and bovine *MSTN* mutant. As a result, ten types of ENU-induced mutations were identified including a nonsense mutation in the investigated region with HRM analysis. In addition, the average mutation frequency in fugu *Mstn* gene was 1 mutant per 297 kb, which is similar to values calculated for zebrafish and medaka TILLING libraries.

**Conclusions:**

These results demonstrate that the TILLING method in fugu was established. We anticipate that this TILLING approach can be used to generate a wide range of mutant alleles, and be applicable to many farmed fish that can be chemically mutagenized.

## Background

Mutations are the basis of genetic variation and mutant populations are indispensable genetic resources in all organisms. This variation can be either naturally occurring or, in plants, animals and lower organisms, induced by chemical or physical treatments. Mutation induction, for example, has played an important role in the genetic improvement of crop species that are of economic significance.

In breeding of aquacultural fish species, it is a final goal to discover and generate the fish with various useful phenotypes for supplying to consumers. Currently genetic breeding is carried out with the search for natural variation in traits such as growth, productivity and disease resistance, and then improved strains are produced by genetic selection or marker-assisted breeding approaches [1]. Thus the success of fish breeding solely depends on the mutations pre-existing in the focal population. The rate of natural spontaneous mutations in fish species is generally lower than 1.0×10^-6^ at specific loci. On the other hand, mutation frequency caused by radiation and chemical mutagenesis range from 1.0×10^-3^ to 3.9×10^-3^ at specific loci when *in vivo* spermatogonial treatments were carried out in zebrafish and medaka [2-4]. Given the high frequency of the mutation ratio, it is reasonable to consider that the chemical mutagenesis will be an efficient way to produce new mutants for future genetic improvement in aquacultural species.

TILLING (Targeting Induced Local Lesions INGenomes) is a reverse genetics strategy that identifies induced mutations in specific genes of interest in chemically mutagenized populations with high-throughput discovery technologies. TILLING, first described in 2000 for mutation detection in *Arabidopsis*[5], is now used in a wide range of plants including soybean [6], rice [7], barley [8] and maize [9] as well as for animal model systems, including *Drosophila*[10], *Caenorhabditis elegans*[11], rat [12], medaka [13], zebrafish [14] and for the discovery of naturally occurring polymorphisms in humans [15].

The first step in TILLING is chemical mutagenesis. In plants, mutations have been induced by using ethylmethane sulfonate (EMS) and methylnitrosourea (MNU) [5,7]. In animals, EMS is used in many invertebrate species for mutagenesis, whereas *N*-ethyl-*N*-nitrosourea (ENU) is used for males of vertebrates, and is the most widely employed method [16]. ENU acts as an alkylating agent and transfers its ethyl group to nucleophilic nitrogen or oxygen sites on deoxyribonucleotides, leading to base mismatch during DNA replication. Single-base substitutions that resemble natural spontaneous mutations [17,18], are mainly induced by ENU treatment and all genes are mutated at random [19]. Because this protocol induces mutations in germ-line stem cells, mutations are transmitted at the rate for at least several months after the treatment. Also, the mutations are induced with premeiotic ENU treatment, so that the initial mutation, usually an ethylation of a base on one DNA strand, is fixed by DNA replication prior to production of differentiated sperm, leading to non-mosaic offspring in the next generation [16]. On the other hand, it has been recently reported that some point mutations were induced by postmeiotic mutagenesis in *Ctenopharyngodon idellus* (grass carp) with ENU for mature sperm [20]. In zebrafish, the postmeiotic ENU mutagenesis resulted in mosaic progeny, but produced a 10 fold increase in the frequency of induced mutations [21]. This protocol in zebrafish has been also shown to induce mutations such as deletions and translocations triggered by chromosomal rearrangements [18]. These results indicate that postmeitotic ENU treatment of male germ cells can induce a variety of mutations from point mutations to deletions and translocations.

In zebrafish and medaka, which are model fish, mutations are introduced in spermatogonia by soaking founder fish in ENU solution [13,22]. However, this protocol is difficult to apply to many aquaculture species including fugu, as the body size of these fish is markedly larger than that of medaka and zebrafish. Thus alternative protocols are required for fish with a large body size.

After the ENU treatment, a large F_1_ population is generated to choose the mutations for purpose from many random heterozygous mutations in their genomes. As the next step in TILLING, the genomic DNA from these animals containing induced mutations is screened with discovery technologies. Sequencing is considered the standard for DNA-based mutation detection because it reveals the exact location and the type of mutations. Taniguchi et al. employed a direct sequencing approach even for the prescreening step [13]. CEL I nuclease-mediated screening of heteroduplexes are widely carried out to detect mutations in TILLING [23,24]. Both methods are effective, but the costs are substantial and it takes a relatively long time for the screening. Recently, high resolution melting (HRM) analysis has succeeded to detect point mutations in plant populations [25], medaka TILLING library [26] and human disease [27]. HRM analysis makes use of characteristics in DNA as follows; the thermal stability of a DNA fragment is determined by its base sequence. When the DNA fragment contains an altered sequence, the duplex stability is changed, leading to different melting behavior. Thus changes in the sequence within the DNA fragment such as point mutation can be detected by the melting analysis.

To apply TILLING technology for breeding practices, it is critical to select a proper target gene in which mutations are likely to cause useful phenotypes such as rapid growth and disease resistance. One of the growth-related factors, Myostatin (Mstn) is a member of the transforming growth factor-β (TGF-β) superfamily, and functions as a negative regulator of skeletal muscle mass [28]. Naturally occurring mutations in the *Mstn* gene have been identified in double-muscling cattle breeds [29,30], mice [28], dogs [31] and even humans [32]. Recently, we have demonstrated that the antagonistic role of Mstn against muscle growth is conserved from mammals to fish species [33]. Indeed *mstn*-deficient medaka (*mstnC315Y*), which were generated by the TILLING method, show a significant increase in body weight in comparison with wild-type medaka, due to over-growth of muscles. Therefore, *Mstn* is a promising target gene for mutation breeding in fishes.

Here, in order to initiate targeted mutation breeding in fish improvement, we searched for efficient methods for TILLING combining ENU mutagenesis with HRM analysis in *Takifugu rubripes*, tiger pufferfish (fugu). Fugu males were treated with ENU via intraperitoneal injection each week for three weeks, and the sperm collected from the founder fish was used for *in vitro* fertilization to obtain the progeny. We then isolated genomic DNA from the progeny, and carried out HRM analysis targeting the exon 3 of fugu *Mstn* gene. Our screening successfully identified ten mutations including one nonsense mutation in fugu *Mstn* gene. The mutation frequency in fugu *Mstn* gene was similar to that identified in the medaka TILLING library, demonstrating that we were able to establish an effective protocol of the TILLING method in fugu. This protocol is likely to be applicable to various species of aquacultured fish.

## Results

### Development of the ENU mutagenesis protocol for fugu TILLING

**Figure 1:**
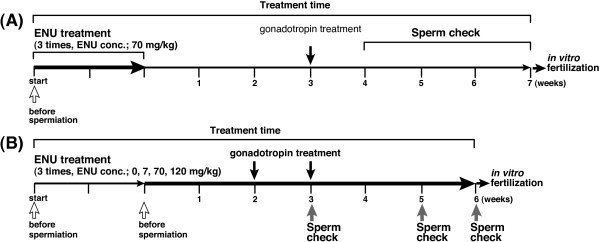
**Scheme design of the ENU mutagenesis in fugu. A)** The number of ENU treatments in fugu. The number and timing of ENU injections was examined. ENU (concentration: 70 mg/kg body weight of fugu) was injected 3 times at weekly intervals into the intraperitoneal of fugu males at the stage before spermiation. **B)** Treatment with various ENU concentrations, and timing of gonadotrophin (HCG/SPH) treatment and sperm collection. Founder fish were treated with three ENU concentrations (7, 70, 120 mg/kg body weight of fugu) at the stage before spermiation. As a control, the fish were treated with buffer (0 mg/kg body weight of fugu) instead of ENU. HCG/SPH was injected into the fugu intramuscularly at 2 or 3 weeks after the last ENU injection. Sperm was collected at 3, 5, and 6 weeks after the last ENU injection.

In order to develop a protocol for ENU mutagenesis in fugu, we performed intraperitoneal injections of ENU, referring to the method used for mouse mutagenesis [34]. Previous study has shown that inheritable point mutations are induced by ENU treatment at the spermatogonia stage in mouse [35]. Based on the report, fugu males were injected with ENU at a dose of 70 mg/kg body weight, 3 times at weekly intervals at the stage before spermiation when early stages of spermatogenesis were progressing (Figure 1A). The injected fish began to die at 4 to 6 weeks after the last ENU treatment (average survival rate: 71%). The surviving fish were treated with the hormone, gonadotropin, to trigger sperm production and spermiation. Their sperm was collected for fertilization and cryopreservation during 6 to 7 weeks after the ENU treatment. No fish survived more than 9 weeks after the last ENU treatment. We then performed *in vitro* fertilization using the collected sperm to obtain mutagenized F_1_ fry. The average fertilization ratio was 89%, which is comparable to the ratio of non-mutagenized sperm. Although the hatching ratio was low (average: 23%), most of the hatched fry had a normal appearance without teratogenesis. Therefore, we determined the method for the ENU mutagenesis in fugu as follows: the founder fish are treated with the 70 mg/kg of ENU concentration 3 times at weekly intervals during the early mature stage to the late maturation stage in spermatogenesis of the fish.

ENU treatment in TILLING method has been shown to effectively produce a lot of mutations. In mouse, ENU induces mutations at a relatively high rate with a dose dependency [36]. Next, we examined whether various ENU concentrations were effective on viability of the founder fish and their progeny (Figure 1B). The fish were injected intraperitoneally with ENU at a dose of 7, 70 or 120 mg/kg body weight, and with the buffer prepared for dissolving ENU as the control (0 mg/kg body weight) to remove the effect of injection on the fish. We examined the number of surviving founder fish after treatment with each ENU concentration. Although almost all fish with ENU in a dose of 7, 70 mg/kg (survival rates: 100% and 92%, respectively) survived at 4 weeks after the last ENU treatment, the fish with ENU in a dose of 120 mg/kg (survival rate: 67%) began to die at that time. The fish, which were collected sperm at 8 weeks after the last ENU treatment, survived till 5 months after the ENU experiments. Final survival rates of each ENU concentration were as follows: 7 mg/kg, 100%; 70 mg/kg, 50%; 120 mg/kg, 33%. After gonadotropin treatment in each family, the collected sperm was used for *in vitro* fertilization. The fertilization ratio (average: 72%) and the hatching ratio (average: 54%) in each cross were similar to those of control (67% and 53%, respectively; Table 1). More than ten thousand hatched fry of fugu were obtained in each family. They revealed that the fertilization and hatching ratios were unaffected by the dosage of ENU when the concentration of ENU was varied from 7 mg/kg to 120 mg/kg.

**Table 1:** Effects of various ENU concentrations on fertilization and hatching ratios of fugu

**ENU conc. (mg/kg body weight)**	**Fertilization ratio (%)**	**Hatching ratio (%)**
0	67	53
7	69	56
70	68	54
70	73	58
70	76	61
120	75	40

*In vitro* fertilization was carried out by using eggs from wild-type fugu and the sperm treated with ENU concentrations as described above. 100 of fertilized eggs in each family were counted at the 4 cell stage for fertilization ratio, while at the melanophore appearing stage for the hatching ratio. These ratios were calculated according to Methods. Founder fish treated with buffer was used as the control (ENU conc., 0 mg/kg).

In the course of the above experiment, we noted that the ENU-treated founder fish did not spermiate without the gonadotropin treatment injection. Then, we investigated the timing of the gonadotropin treatment to maximize the amount of sperm to collect (Figure 1B). Gonadotropin treatment was performed with the fish at 2 or 3 weeks after the last ENU treatment, which was the stage before the fish began to die as described above. To determine the time period for sperm collection after the gonadotropin treatment, we counted the number of spermiated fish at 3, 5 and 6 weeks after the last ENU treatment, and measured the volume of sperm (Table 2). In the gonadotropin treatment at 2 and 3 weeks after the last ENU treatment, sperm was collected at both 5 and 6 weeks, while fish did not spermiate at 3 weeks after the last ENU treatment. The frequency of spermiated fish was higher in the gonadotropin treatment at 3 weeks than at 2 weeks after the last ENU treatment. There was no noticeable difference between the volume of sperm in each week after the gonadotropin treatment. In the control without the gonadotropin treatment, the fish did not spermiate until 5 weeks after the last ENU treatment, and produce a very small amount of sperm at 6 weeks. The volumes of all sperm collected under these conditions were sufficient for cryopreservation, except for the fish without hormonal treatment. From the results, we fixed the method as follows: the gonadotropin treatment for the founder was carried out at 3 weeks after the last ENU treatment, and then the sperm from the fish was collected at 3 weeks after the gonadotropin treatment.

**Table 2:** Effects of gonadotropin treatment on timing of sperm collection and the sperm volume in fugu

		**Period after the last ENU treatment**					
		**3 weeks**		**5 weeks**		**6 weeks**	
	**ENU conc. (mg/kg)**	No. of fish from which sperm was collected/No. of fish examined (%)*	**Sperm volume (ml)**	No. of fish from which sperm was collected/No. of fish examined (%)*	**Sperm volume (ml)**	No. of fish from which sperm was collected/No. of fish examined (%)*	**Sperm volume (ml)**
Gonadotropin treatment (2 weeks after the last ENU treatment)	70	0/4 (0)	0	2/4 (50)	5-15	3/4 (75)	4-10
Gonadotropin treatment (3 weeks after the last ENU treatment)	0	-	-	2/2 (100)	4-9	1/1 (100)	6
	7	-	-	3/3 (100)	7.5-30	3/3 (100)	8-10
	70	-	-	3/3 (100)	10-17.5	3/3 (100)	5-7
	120	-	-	0/2 (0)	0	1/1 (100)	3
Non-gonadotropin treatment (control)	70	0/3 (0)	0		0	3/3 (100)	0.2-2

(%)* reveals ratio of fish which were able to collect the ENU-mutagenized sperm to the examined fish.

### Target gene for generation of a fugu mutant with TILLING

**Figure 2:**
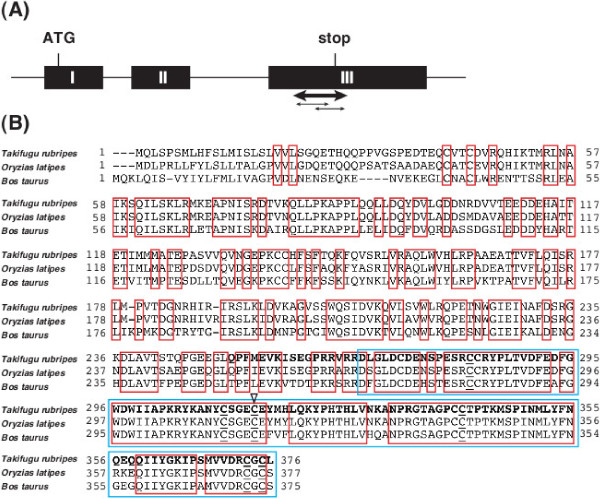
**Fugu Mstn profiles. A)** Genome structure of fugu *Mstn* gene (1905 bp). Black boxes, exons (with the Roman numeral representing the number of exons) and bars, introns. **B)** Sequence alignment of fugu [GenBank, ADT89782], medaka [GenBank, NP_001188428] and bovine [GenBank, NP_001001525] Mstn protein. Amino acids conserved across the three species are outlined in red. The amino-acid sequence of fugu Mstn shows 83.8% and 60.1% identity to that of medaka and bovine, respectively. The C-terminal peptide, which is considered to be significant for the function, is outlined in light blue. The underlined cysteines are essential for the activity of Mstn in vertebrate. The fugu C-terminal region has an 88.1% identity to medaka and bovine Mstn. Black triangle, the amino acid responsible for the loss-of-function phenotype in medaka and bovine. Bidirectional bold and thin arrows **(A)** and bold characters **(B)**, the region examined by the HRM analysis in this study.

Our long-term goal is to generate a fugu line that exhibits a useful phenotype for the aquaculture industry by TILLING and to apply this technology to a variety of aquacultured species. Recently, we have reported that myostatin (*mstn*)-deficient medaka exhibited a double-muscling phenotype with hyperplasia and hypertrophy [33]. There is a high possibility that *Mstn*-deficient fugu exhibits such the phenotype, if a mutation is found in the same position or the significant region of *mstn* gene in medaka. Therefore, we amplified presumable full-length cDNA of fugu *Mstn* using cDNA from the muscle as a template. Sequencing of these products indicated that fugu *Mstn* comprises 3 exons, and encodes a protein consisting of 375 amino acids (Figure 2A). The fugu Mstn amino acid sequence has 83.8% identity to medaka and highly 88.1% identity in the C-terminal active peptide of Mstn (Figure 2B). Search in the fugu genome assembly suggested that fugu has a single copy of this gene.

### Detection of ENU-induced mutations in fugu with HRM analysis and DNA sequencing

**Figure 3:**
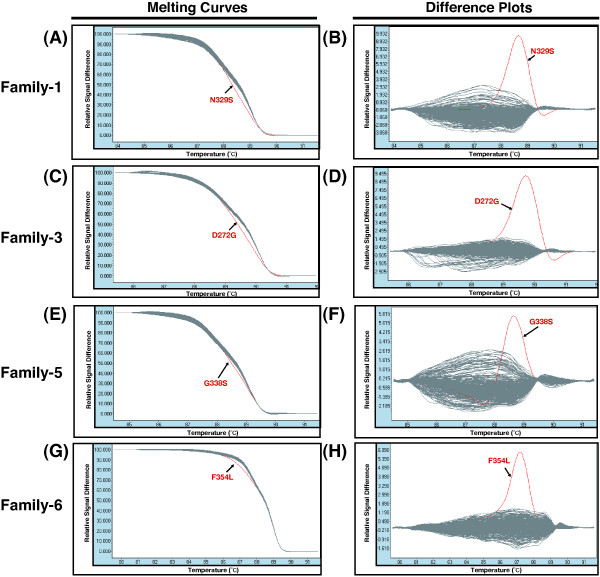
**ENU-induced mutations in fugu *Mstn *gene detected by HRM analysis.** The exon 3 of the *Mstn* gene was examined by HRM analysis with each family of fugu genomic DNA. The region contains the C-terminal peptide of the fugu Mstn protein. The family numbers mean the line of the hatched fry generated by *in vitro* fertilization (see Table 3). Left side of figures, the high-resolution melting curve **(A, C, E, G)**, and right side of the figures, the substitution plots **(B, D, F, H)**. X-axis and Y-axis, temperature (°C) and relative signal difference, respectively. Red lines and black arrows, the mutations screened by the HRM analysis.

**Figure 4:**
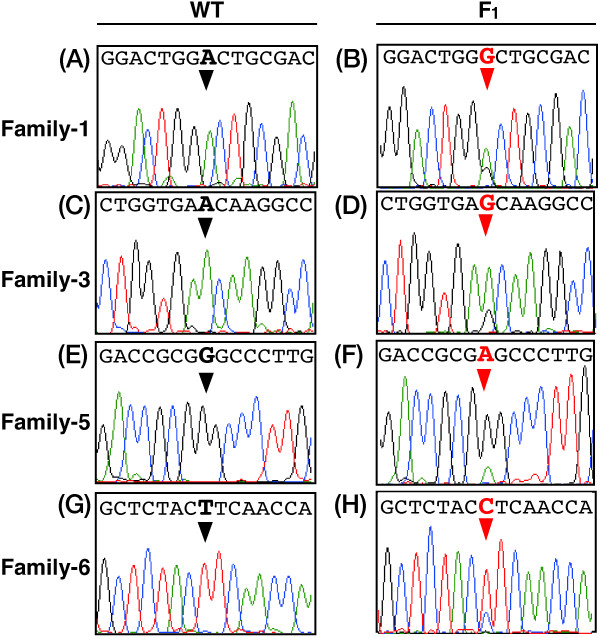
**ENU-induced point mutations in fugu *Mstn *gene identified by direct sequencing.** The PCR products from fugu genomic DNA screened by the HRM analysis were investigated by direct sequencing. Left side **(A, C, E, G)** and right side **(B, D, F, H)** of figures, the sequence traces of wild-type fugu (WT) and the fugu mutant (F_1_). Lines in green, red, black and blue; adenine, thymine, guanine and cytosine, respectively. A nucleotide in the germ cell in the founder fish (black triangles) is replaced by another nucleotide due to the ENU-treatment (red triangles), resulting in an offspring that is heterozygous at the site. The numbers mean the line of the hatched fry generated by *in vitro* fertilization (see Table 3).

Since TILLING can identify mutations in the heterozygous condition, it is possible to screen using the F_1_ offspring of ENU mutagenesis. We performed the HRM analysis to screen for ENU-induced mutations in the C-terminal region of fugu *Mstn* gene, since the region is highly conserved among vertebrates (Figure 2), and is responsible for the function of Mstn [37]. To screen correctly and quickly a large amount of genomic DNA, efficient protocols for HRM analysis were explored with a DNA automatic dispensing machine and a detection device for 384 samples. Genomic DNA was extracted from 1,056 fry obtained from in *vitro* fertilization with the ENU-treated sperm. These DNA were subjected to the nested PCR with some primers of the fugu Mstn (see Methods) and two amplicons Figure 2A (268 bp and 281 bp) were used for HRM analysis. Examples of the melting curves and the differentiation plots in fugu *Mstn* gene are shown in Figure 3. The mutation candidates were clearly indicated in each family (Figure 3, red lines). The amplicons selected as positive samples were subjected to direct sequencing in order to confirm whether they contain mutations. Overlapping peaks in the DNA sequence traces revealed that an F_1_ individual carries a heterozygous point mutation (Figure 4), while this mutation was not observed in the rest of the siblings as well as in both parents. The results demonstrate that the mutations were induced by ENU treatment in fugu.

As shown in Table 3, ten types of mutations were detected by HRM analysis and then confirmed by direct sequencing (nonsense, 1; missense, 7; silent, 2). Also, it was found that all the ENU-induced mutations in fugu were point mutations. The number of the induced mutations was an average of 1 to 2 in each family treated with various ENU concentrations (7, 70, 120 mg/kg body weight). When the effect of injection into fugu on the mutations was examined in the control (0 mg/kg body weight), no mutations were recognized. This indicates that the number of induced mutations does not depend on the concentration of ENU within the range of 7 to 120 mg/kg. Consistent with this, the dose-dependent effects were not observed on the fertilization and the hatching ratios (Table 2).

**Table 3:** Summary of fugu MSTN mutations detected by HRM analysis

**Family no.**	**ENU conc. (mg/kg body weight)**	**DNA sequence**	**Amino acid substitution**
1	7	CTGGGACTGG(A > G)CTGCGACGAG	D272G
1	7	TTGCAGAAAT(A > G)CCCGCACACC	Y322C
2	70	gCAACCTTTC(A > G)TGGAGGTGAA	M254V
3	70	GAGACCTGGG(A > G)CTGGACTGCG	G270G
3	70	CACCTGGTGA(A > G)CAAGGCCAAC	N329S
4	70	AGTGTGAGTA(C > A)ATGCACTTGC	Y316X
5	70	CGCACACCCA(C > T)CTGGTGAACA	H326H
5	70	AGGGACCGCG(G > A)GCCCTTGCTG	G338S
6	120	CATGCTCTAC(T > C)TCAACCAAGA	F354L
6	120	CTCTACTTCA(A > G)CCAAGAACAG	N355S

(A > B): A is the nucleotide of wild-type and B is the nucleotide of the mutant. X indicates a stop codon. There were ten types of mutation in the investigated region with HRM analysis (nonsense, 1; missense, 7; silent, 2). The nonsense mutation is underlined.

### Identification of Mstn-deficient mutation in fugu

**Figure 5:**
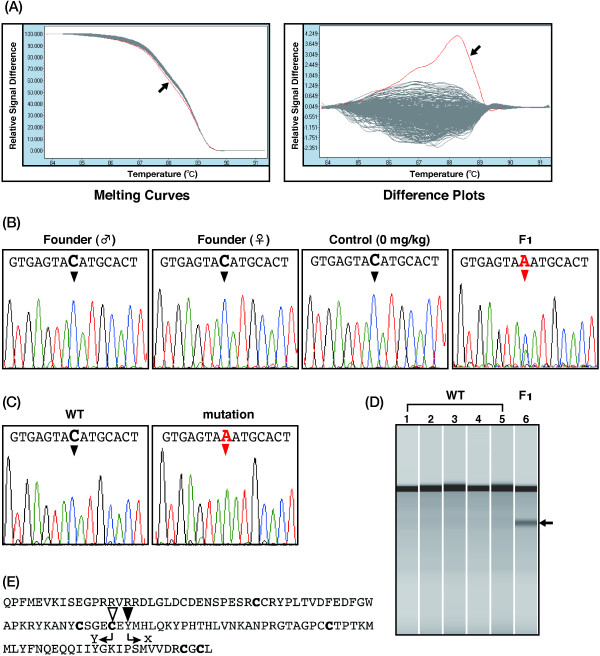
**Identification of the ENU-induced nonsense mutation in fugu TILLING. A)** The high-resolution melting curve (left) and the substitution plot (right). Exon 3 of the *Mstn* gene, which contains the C-terminal region of the Mstn protein, was examined by HRM analysis using the fugu family 4 (see Table 3). Red curve, the deviated curve due to mutation. **B)** Direct sequencing for the selected mutations. The selected genomic DNA with the HRM analysis was investigated by direct sequencing. Lines in green, red, black and blue; adenine, thymine, guanine and cytosine, respectively. The sequence traces of the fugu parents (Founder-♂ and -♀), control (Control), and the ENU-induced mutant (F_1_) are illustrated in order from left to right. Cytosine in the parents (black triangles) is substituted to adenine in the mutant (red triangle), resulting in the change from tyrosine to a stop codon at amino acid 316. **C, D)** Confirmation of the nonsense mutation in fugu TILLING. PCR amplicons were cloned and sequenced to confirm the substitution of the nucleotide **(C)**. The sequence traces of the cloned wild-type and the mutant DNA are illustrated in left (WT) and right (mutation), respectively. Cytosine to adenine nucleotide transition was recognized. The cleavage product was detected with CEL I assay in the mutant DNA (**D**, lane 6), but not in wild-type DNA (**D**, lanes 1–5). Black arrow, the cleavage product. **E)** Nonsense mutation in the C-terminal region of fugu Mstn. The ENU-induced point mutation in fugu TILLING caused the substitution to a stop codon in the C-terminal region. Bold characteristics, six cysteines which are essential for the Mstn conformation. X, a stop codon. Black and white triangles, amino-acid change from tyrosine to a stop codon in fugu Mstn; the change from cysteine to tyrosine in medaka Mstn responsible for the phenotype, respectively.

The nonsense mutation we discovered changes a cytosine to an adenine in exon 3 of fugu *Mstn* converting a tyrosine codon to a stop codon in a fry from the family 4 (Figure 5A, B, Table 3). While the fry was heterozygous at this site, its parents and control fish were homozygous (Figure 5B), suggesting that the mutation was induced by the ENU treatment. DNA sequencing of the amplicon cloned into a plasmid vector confirmed the presence of this mutation (Figure 5C). The nonsense mutation was also examined with CEL I assay (Figure 5D). The cleavage product was detected in the CEL I reactions of the mutant DNA (lane 6), but they were not in the reactions of wild-type DNA (lanes 1–5). The mutation likely results in the loss-of-function of Mstn in fugu, as a critical part of the amino acid chain, the C-terminal peptide, are no longer created due to the stop codon.

### ENU-induced mutation ratio in fugu

The mutation ratio in the mutagenesis is a key point to produce the mutants effectively. Therefore, the ratio was calculated using data detected in fugu *Mstn* gene by HRM analysis. We screened about 3.0 Mb in total and identified 10 independent mutations (Table 3). Therefore, the ENU-induced mutation ratio for fugu *Mstn* gene was one substitution per 297 kb.

## Discussion

### Establishment of fugu TILLING

**Figure 6:**
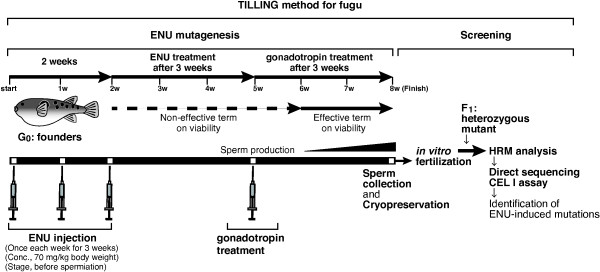
**Establishment for the fugu TILLING method.** Mutagenesis was carried out with injection of ENU (conc., 70 mg/kg body weight) into founder fugu 3 times at weekly intervals. Half of the founder fish died around 6 weeks after the last ENU treatment. Sperm was not obtained from the founder until 5 weeks after the last ENU treatment. To collect the sperm from the founder, gonadotrophin (HCG/SPH) was injected once 3 weeks after the last ENU treatment. The sperm was collected 3 weeks after the hormone treatment and then cryopreserved for a long-term TILLING resource. For screening strategy, the hatched fry were obtained *in vitro* fertilization with the collected sperm and eggs from wild-type female. Genomic DNA extracted from the fry was used to screen the induced mutations in *Mstn* gene with HRM analysis. The screened mutations were confirmed by direct sequencing and CEL I assay. These approaches demonstrate that the TILLING method was established in fugu.

Schematic model for fugu TILLING method is summarized in Figure 6 as follows: fugu males are treated with ENU 3 times at weekly intervals at the stage before spermiation and then treated with gonadotropin at 3 weeks after the last ENU treatment to facilitate the sperm production and spermiation. The sperm from the founder fish was collected at 3 weeks after the gonadotropin treatment and used for *in vitro* fertilization to obtain hatched fry. The genomic DNA extracted from the fry was subjected to PCR and the amplicons were used to screen ENU-induced mutations with HRM in a fugu gene of interest. Our study firstly demonstrated that TILLING technology is applicable to aquacultured fish. Interestingly, the TILLING approach in fugu could be used in a similar way as in plants [38]. We can treat them like plant seeds derived from mutagenized ancestor, because sufficient eggs and sperm to obtain a large amount of F_1_ progeny are prepared for a single pair of fugu female and male. Furthermore, mutations induced by TILLING in fugu can be readily distinguished from endogenous SNP, since a single female and male pair is used for the cross. On the contrary, it has been pointed out that non-predictable frequency of endogenous SNP in a mutagenized bank derived from multiple founders is an obstacle for the screening in zebrafish and medaka [13,22]. TILLING can be considered as an appropriate method for many aquacultured fish producing a large number of eggs.

Recently, *in vitro* ENU mutagenesis of postmeiotic sperm was performed in grass carp [20]. In their study, the induced mutations were investigated using growth-related genes (*Igf-2a*, *Igf-2b*, *Mstn-1*, *Mstn-2*, *Fst-1* and *Fst-2*) and the estimated mutation ratio was around 0.41%. However, the mutations are apparently mosaic in F_1_ individuals. Mosaicism of the induced mutation was also reported in zebrafish when postmeiotic sperm was mutagenized [21]. This situation makes recovery of the mutant line inefficient in contrast to the case of non-mosaic mutation induced by ENU-treatment of spermatogonia [21]. The mosaicism in F_1_ individuals and resulting non-Mendelian transmission from F_1_ to F_2_ generations hinder some screening protocols. In addition, large-scale rearrangements of the genomic structure such as translocation of genomic segments have been reported following the mutagenesis of postmeiotic sperm [18]. Therefore, the suitability of the *in vitro* ENU mutagenesis of postmeiotic sperm for mutation breeding in fish improvement remains to be verified. Although the *in vitro* ENU treatment is more convenient than the *in vivo* methods used in fugu and other model animals, it will be required to examine whether the induced mutations will be inherited by the progeny in the mutants.

In TILLING, high efficiency for ENU-induced mutations is required to obtain various phenotypes in gene of interest. A previous report indicated that the induced mutation frequency increased with ENU dose dependently in mouse [36]. In this study, there was no difference in the number of surviving fish, fertilization and hatching ratios in ENU treatment with 7, 70 mg/kg (Table 2). ENU treatment with 120 mg/kg affected slightly on the number of surviving treated fugu. However, the number of the induced mutations did not tend to increase in each ENU concentration between 7 mg/kg and 120 mg/kg (Table 3). These results suggested that each ENU concentration in the method may become saturation in premeiotic sperm (spermatogonia and spermatocyte cells) of fugu.

### Identification of fugu Mstn mutant

It has been reported that loss of the Mstn function in medaka and mice causes double-muscling phenotype with both hyperplasia and hypertrophy [28,33]. In the present study, ENU-induced mutations in fugu Mstn were confirmed and in particular, the nonsense mutation (Y316X) was identified at C-terminal region of the peptide. The *mstn* mutant medaka (*mstnC315Y*) made by using the TILLING method [13] corresponded to a naturally occurring mutation (C313Y) in double-muscled Piedmontese cattle [29] (Figure 2B). The cysteine residue at the 315-position exists in C-terminal region of the Mstn peptide (Figures 2B and 5D) and is known to be one of the important amino acid residues involved in forming the cysteine knot structure in members of the TGF-β superfamily [37]. The structure functions as a homodimer to elicit a biological function [39]. Therefore, we speculate that the structural formation composed of six essential cysteines in fugu Mstn will be non functional by the truncated form of the peptide resulting from a point mutation at 317 of Tyr. Eventually, the mutation will be conceivable to cause loss of the Mstn function in fugu *Mstn* mutant. The double-muscling phenotype in medaka and bovine could be similarly expected for an *Mstn*-deficient mutant fugu.

### Development of HRM analysis

For screening, there are a variety of mutation discovery technologies such as direct DNA sequencing, CEL I assay and HRM analysis [13,23,26]. We introduced the HRM analysis for screening system in fugu because there is no albino mutant in fugu for specific locus test (SLT) and the analysis has recently succeeded to detect point mutations as a screening step for the TILLING approach [25,26]. But, there was room for improvement with respect to the cost and variability of output data. In this study, we have developed a system of HRM analysis as follows: cost-cutting with fluorescent intercalator; SYTO 9, introduction of the nested PCR for precision enhancement and avoidance of variability, speed-up with 384 samples treatment in a single experiment, and high uniformity of dispensed volume and removal of human error by using the automatic dispense device. As a result of the improvement, some mutations could be detected efficiently in the analysis (Figure 4 and Table 3). It is difficult for HRM analysis to investigate mutations with a long amplicon in comparison with the other methods, but the acquisition of melting profiles in HRM is not dependent on the location and number of mutations present in the analyzed fragment. It has been compared about the mutations detected by the screening approaches. The results indicate that HRM analysis is useful as resequencing in medaka [26] and more efficient than CEL I assay in mammals [40]. Taken together, these demonstrate that HRM analysis is a reliable high-throughput approach for screening ENU-mutagenized individuals in potentially various species.

### Mutation ratios in fugu TILLING

The mutation ratio in ENU mutagenesis has been calculated in a SLT using the albino mutant in zebrafish and medaka [21,41]. The characteristics of the albino mutant in fish reveal mainly red eyes and one of the responsible genes is tyrosinase. In some aquacultured fish with albino mutants, it may be easy for these ENU-treated fish to examine the mutation ratio with SLT. However, albino mutants are not found in nature in all animal species.

Instead of SLT, the mutation ratio was calculated using base-pairs screened and the number of mutations detected in fugu *Mstn* gene by HRM analysis (Table 3). The frequency in fugu (1 mutation per 297 kb) is similar to the mutation frequency in medaka (1 mutant per 345 kb) and in zebrafish (1 mutant per 235 kb), which were calculated by resequencing data [13,42]. This suggests that ENU mutagenesis in fugu was efficiently performed in our way and the protocol detailed here will potentially be useful for constructing a TILLING library.

CG to AT (or reverse) changes are often detected in HRM analysis, but the endogenous SNPs are also detected as the same changes and give the most significant difference in this analysis. In our study, there were no endogenous SNPs in the C-terminal region of the *Mstn* gene in both the investigated family of fugu and their parents (data not shown). Although G > C, C > G, T > A, A > T mutations were not observed in the 10 mutations detected in the *Mstn* gene (Table 3), it was reasonable results since the appearance frequency of their mutations are also rare in human genome [43].

### Generation of an Mstn-deficient mutant in fugu

Our next step will be to generate the *Mstn* mutant for the purpose of serving the aquaculture industry. One of the key components for a successful TILLING project is a near-perfect ability to recover valuable mutations once they have been found. For the recovery, TILLING libraries can either be ‘living’ or cryopreserved as suggested by Moens et al. [22]. A living library is one in which mutagenized F_1_ fish are held in pools in tanks while their fin-tip genomic DNA is screened. A cryopreserved library is one in which each genomic DNA sample corresponds to vials of frozen sperm that can be used in an *in vitro* fertilization to generate F_2_ heterozygotes. As a large amount of sperm can be stored from a single adult male of aquacultured fish, in general, the cryopreserved library is more desirable than ‘living’ library. However, generation time of many aquacultured fish including fugu is more than one year, making the construction of the cryopreserved library time-consuming. We are currently generating an *Mstn*-deficient fugu with ‘living’ library as the next step.

## Conclusions

In the present study, the fugu TILLING method was established from the results in both ENU mutagenesis and a mutation search technology, HRM analysis. This is a first report that TILLING is an applicable method for aquacultured fish. The approach for the fish species might play an important role in producing a fish, which exhibits a valuable phenotype (e.g., rapid growth, disease resistance) for the aquaculture industry. We hope that the TILLING technique will be an alternative to the classic selection approach for improving traits in aquacultured fish species.

## Methods

### Ethics statement

Fisheries Research Agency and The University of Tokyo rule that ethic approval is not necessary for study of fish. Nevertheless, this project was conducted in accordance with the Regulation for Animal Experiments in the Fisheries Research Agency, which stipulated in the Fundamental Guidelines for Proper Conduct of Animal Experiments and Related Activities in Research Institutions (Ministry of Agriculture, Forestry and Fisheries of Japan), and under the supervision of the Animal Care and Use Committee for the National Research Institute of Aquaculture. In this study, ENU and hormone injections for fugu were carried out under 0.05% anesthetic agent (2-phenoxy-ethanol) and hatching fry of fugu were harvested after they were soaked in sea water containing ice. All efforts were made to minimize suffering.

### Experimental fish and eggs for *in vitro* fertilization

50 fugu males (body weight: around 1 kg) were purchased from a commercial supplier (Shihou Suisan, Minami-ise, Mie). The fish were kept at the Nansei station of National Research Institute until obtaining sperm from the ENU-treated fish. Three-year old fugu females (body weight: around 2–3 kg) were cultivated in 30–50 ton of seawater tanks at the Yashima station of National Center. Two independent fugu females were used to obtain eggs for *in vitro* fertilization. Mainly, the fertilization and harvest of the hatched fry were performed at the Yashima station. A part of the fertilized-eggs were packed into seawater without air and sent to Fisheries Laboratory attached to the University of Tokyo. The fertilized eggs were incubated at 18°C during 8 to 10 days until harvest of the hatched fry.

### Mutagenesis with ENU

All working solutions of ENU (Conc.: 11 mg/ml, Sigma-Ardrich, St. Louis, MO USA) were freshly prepared following the methods described by Taniguchi et al. [13]. ENU was prepared at three concentrations (7, 70, 120 mg per kg, body weight of fugu). For ENU mutagenesis, fugu males (around 1 kg of body weight) were used and injected into the intraperitoneal with the ENU concentrations prepared for 10 mM sodium phosphate buffer (pH 6.3). The injections were repeated 3 times at weekly intervals to the founder fish. After the last ENU injection, the fish were injected intramuscularly with human chorionic gonadotropin (HCG, 500 IU/kg body weight) or salmon pituitary homogenate (SPH, 10 mg/kg body weight), consisting of acetone-dried chum salmon pituitary homogenized in Ringer, was added to HCG because of increasing the success rate of spermiation in ENU-treated males. After the hormone treatment, the sperm was collected from each fish.

### Cryopreservation of sperm

The freshly-collected sperm from each fugu male was suspended at the rate of 1 to 4 in freezing diluents [10% dimethyl sulphoxide (Sigma-Aldrich Co., Tokyo, Japan) in 90% fetal bovine serum (Thermo Scientific Co., Tokyo, Japan)] and cryopreserved as described by Ohta et al. [44]. The amount of sperm held in each capillary (0.5 ml vol.) was enough to fertilize more than twenty thousand eggs.

### *In vitro* fertilization

About one million unfertilized eggs were squeezed out from a fugu female, which was induced ovulation by administration of an LHRH analogue (des Gly10 [D-Ala6]-LHRH, 400 μg/kg body weight). Fresh sperm, obtained from each fugu family line, was used in this method, and the sperm activity was examined under a light microscope. 0.4 ml of the sperm was added to 12 g of the eggs (around twenty thousand eggs), and these were mixed well. After adding seawater to activate the sperm for fertilization, the eggs were immediately mixed and stood for 5 minutes. The eggs were washed 3 times with new seawater to remove the extra sperm after discarding the remaining seawater. The fertilized eggs were incubated for 4 to 6 hours at 18°C. The eggs were moved to a hatching tank with running water and kept at 18°C until they hatched. At 8 to 10 days after fertilization, around ten thousand hatched fry were obtained in each family. These fry were harvested in a 50 ml tube containing 99.5% ethanol (EtOH) and stored at −30°C.

The fertilization ratio was determined at the four-cell stage with Sera solution (EtOH 6: formalin 3: acetic acid 1), which makes the fertilized eggs transparent. In addition, one hundred eggs were counted at melanophore stage in the fertilized eggs of each cross, and then incubated at 18°C until hatching. The hatching ratio was calculated as the number of the hatched fry per 100 fertilized eggs.

### Cloning of fugu *Mstn* gene

RNA was extracted from wild-type fugu skeletal muscle by RNeasy® Mini Kit (QIAGEN Sciences, MD, USA) according to the manufacturer’s instructions. cDNA was synthesized using PowerScript (BD Biosciences Clontech, Palo Alto, CA, USA). To identify fugu *Mstn* gene orthologs, we used the basic local alignment search tool (BLAST) to search the fugu genome database (http://www.ensembl.org/Takifugu_rubripes/blastview). Fugu *Mstn* cDNA sequences was amplified using a reverse-transcription PCR by gene specific primer pairs flanking the ORF: 5′UTR-F1, 5′-TAGAACGCATTTCCACGTCTTC-3′, 3′UTR-R1: 5′-TGGTTTGAGGCCTCTACTCTGG-3′. Amplicons were cloned into a sequencing vector, and full-length ORF sequences were obtained.

### Identification of mutations by HRM analysis

#### Extraction of genomic DNA

In this study, 1,056 hatched fry of fugu were prepared in each family obtained from *in vitro* fertilization. A single fry was placed into each well of 8-well tubes containing 45 μl of 50 mM NaOH, and incubated at 95°C for 10 min. After incubation, 5 μl of 1 M Tris–HCl (pH 8.0) was added to the lysate immediately for neutralization. These aliquots were centrifuged at 1,100 × g and room temperature for 20 min to remove tissue debris. Then, each 40 μl of supernatant was harvested into 96-deep well plates and stored at −30°C. Although the genomic DNA stocks were crude extract and freeze-thawed repeatedly, we could use the stocks for our assay during 10 months at least.

#### Primers

To find the loss-of-function mutations in the fugu *Mstn* gene, the third exon of the gene was screened using the primers designed by Primer3 programs on the web (source code available at http://bioinfo.ut.ee/primer3-0.4.0/primer3/). The primer sets and amplicon sizes were as follows, the forward and reverse primers #1: 5′-CAGCCATGATCTAACTTCTGCAAG-3′ and 5′-GTTTGAGGCCTCTACTCTGGTTCT-3′, yielding a fragment of 586 bp; the forward and reverse primers #2: 5′-CTCGCTCACATGCATTCTGTC -3′ and 5′-CTTGTTCACCAGGTGGGTGT-3′, yielding a fragment of 268 bp; the forward and reverse primers #3: 5′-AAGCGCTACAAGGCCAACTA -3′ and 5′-AAAGCGCTGGAACTGGTAGA-3′, yielding a fragment of 281 bp. These primers were used for the 1st-PCR (primer #1), and for the 2nd-PCR (primers #2 and #3) [see PCR].

#### PCR

PCR amplicons for HRM were obtained by using a nested-PCR approach. The genomic DNA stocks and all PCR reaction mixtures were dispensed by a laboratory automation system, Biomek®3000 (Beckman Coulter, CA, USA).

The 1st-PCR (PCR_1_) was performed in 96-well PCR plates in 15 μl volumes. Reactions included 2 μl of the genomic DNA solution in 1x KOD-FX PCR buffer (Toyobo, Tokyo, Japan), with 400 μM of deoxynucleotide triphosphate, 0.1 U of KOD-FX polymerase (Toyobo), and 300 nM of each primer. The polymerase was able to increase precise amplicons with crude samples. The plates were centrifuged at 200 × g for 1 min and then PCR was performed in T-Professional Thermocycler (Biometra, Germany). The cycling conditions were set as follows: one cycle of 94°C for 2 min, followed by 40 cycles of 98°C for 10 sec; 60°C for 30 sec; 68°C for 40 sec. The PCR_1_ products were firstly diluted 10 fold for DNA sequencing and then finally 140 fold with sterilized water by using Biomek® 3000. The 140 fold diluted samples were used as a template for second step of PCR reactions.

The 2nd-PCR (PCR_2_) was performed in 384-well PCR plates (Roche Diagnostics, Basel, Switzerland) in 9 μl volumes. Reactions included 2 μl of the diluted PCR_1_ products in 1x Hot-Start Gene Taq PCR buffer (Nippon Gene, Tokyo, Japan), with 200 μM of deoxynucleotide triphosphate, 1.25 μM of SYTO 9 (Molecular Probes, OR, USA) as a fluorescent substance, 0.16 U of Hot-Start Gene Taq polymerase (Nippon Gene), and 200 nM of each primer. The plates were centrifuged at 200 × g for 1 min and then PCR was performed in a 384-well Light Cycler 480 System (Roche Diagnostics). The cycling conditions were set as follows: one cycle of 95°C for 5 min, followed by 25 cycles of 95°C for 10 sec; 60°C for 30 sec; 72°C for 20 sec.

Several samples of the PCR_1_ and PCR_2_ products were examined with a microchip electrophoresis system, MultiNA (Shimadzu, Kyoto, Japan) whether there are the extra major fragments except for the proper fragment or not, because non-specific PCR products reduce the performance of HRM assay.

#### HRM assay

After PCR, the plates were imaged in the 384-well Light Cycler 480 System (Roche Diagnostics). The PCR products in the plates were heated at 0.1°C/second, and fluorescence measurements were collected from 65°C to 98°C. Briefly, background-subtracted fluorescence curves were normalized between 0% and 100%. The curves were viewed on the subtraction plots to magnify differences in the shape of the melting curves. The subtraction plots are useful to cluster the samples into groups. Clustering of the melting curves for genotype identification was carried out using the Light-Cycler software.

A single base-pair change within the probe region causes a significant shift in melting temperature (Tm) of the amplicon. In the TILLING method, all the induced mutations are heterozygous. PCR amplification of heterozygotes followed by heat denaturing and annealing result in the formation of 4 duplexes: two homoduplexes and two heteroduplexes. Each duplex has a characteristic melting temperature, and the sum of all transitions can be detected by melting curve analysis. The heterozygous samples are identified by differences in shape of the melting curve.

1,056 of the genomic DNA in each fugu family were subjected to PCR and two amplicons (268 bp and 281 bp) for screening were examined in HRM. The genomic DNA showing melting curves different from those of the wild-type allele were selected as mutant candidates. As previously reported in the medaka TILLING method [26], a substantial number of SNPs might exist in the fugu genomic DNA used. SNPs were discriminated from true mutations by defining the same type of base change detected in more than two sequencing reactions as an SNP. Moreover, the genomic DNA in the pair of fugu parents extracted from the cut fins was examined by DNA sequencing to confirm whether it was the true mutations or not.

#### Direct sequencing

The positive genomic DNA were screened as candidate mutations by HRM analysis. The PCR_1_ products of the genomic DNA were already diluted 10 fold, and stocked at −30°C [see PCR]. These samples were used as a template for direct DNA sequencing. Sequencing reactions were carried out using a BigDye® terminator ver. 3.1 cycle sequencing kit (Applied Biosystems, CA, USA) and each forward and reverse primers. PCR cycling condition was set as follows: one cycle of 96°C for 60 seconds, followed by 25 cycles of 96°C for 10 seconds; 50°C for 5 seconds; 60°C for 120 seconds. Sequencing products cleaned up by magnetic bead-based purification system (Agencourt CleanSEQ, Beckman Coulter) were run on automated ABI 3130xl DNA analyzers (Applied Biosystems). Sequences were analyzed for the presence of heterozygous mutations using PolyPhred [45] and manual inspection of the mutated positions. All candidate mutations were confirmed with new PCR_1_ products and resequencing read. The PCR_1_ products were treated with an ExoSAP-IT kit (Affymetrix, Ohio, USA) to remove unused primers and nucleotides.

#### CEL I assay

To confirm the presence of induced mutations in the target sequence, CEL I assay was performed with genomic DNA containing the mutations detected by direct sequencing. CEL I enzyme was purified from celery according to Anai et al. [46]. The enzyme activity for each batch of CEL I was determined experimentally using control samples.

PCR was performed in 9 μl volumes, and the primer sets and amplicon size were as follows, the forward and reverse primers: 5′-CTCGCTCACATGCATTCTGTC-3′ and 5′-AAAGCGCTGGAACTGGTAGA-3′, yielding a fragment of 465 bp. Reactions included 2 μl of the diluted PCR_1_ products in 1x Hot-Start Gene Taq PCR buffer (Nippon Gene), with 200 μM of deoxynucleotide triphosphate, 0.16 U of Hot-Start Gene Taq polymerase (Nippon Gene), and 200 nM of each primer. The cycling condition was set as follows: one cycle of 95°C for 5 min, followed by 25 cycles of 95°C for 10 sec; 60°C for 30 sec; 72°C for 40 sec.

Directly following the PCR, heteroduplex was formed by incubating at 98°C for 8 min, 80°C for 20 sec, 70 cycles of 80°C for 7 sec with a decrement of 0.3 sec per cycle. Next, PCR reactions were incubated with 1 μl CEL I enzyme solution in a total volume of 10 μl at 37°C for 60 min. CEL I reactions were stopped by adding 10 mM EDTA. The cleavage products were detected with MultiNA (Shimadzu).

## Abbreviations

TILLING: Targeting Induced Local Lesions In Genomes; ENU: *N*-ethyl-*N*-nitrosourea; HRM: High-resolution melting; Mstn: Myostatin.

## Competing interests

The authors declare that they have no competing interest.

## Authors’ contribution

MK designed and carried out screening of the ENU-induced mutations, wrote the manuscript; TK performed in vitro fertilization, bred fish and counted the fertilized eggs; TI performed in vitro fertilization and bred fish; YY performed in vitro fertilization, bred fish and advised on the interpretation of breeding experiments; YY performed *in vitro* fertilization, counted the fertilized eggs and calculated the fertilization ratio; MF bred fish and helped to count the hatching fry; SC carried out the sequence alignment of Mstn; TU purified the CEL I enzyme and checked the enzyme activity; TM purified the CEL I enzyme; AN carried out extract the genomic DNA and calculated the hatching ratio; YS participated in breeding fish and advised on the interpretation of experiments; KK conceived the study, participated in the design of the screening system and guided the overall project; HO designed and performed injections for the ENU-mutagenesis, and helped to draft the manuscript. All authors have read and approved the final manuscript.
